# Statin use and all-cause mortality in critically ill ischemic stroke patients: a time-dependent Cox analysis with external validation highlighting enhanced survival benefits in dementia subgroups

**DOI:** 10.3389/fnagi.2026.1866416

**Published:** 2026-09-15

**Authors:** Danyu Li, Ruixin Liu, Hui Xu, Hongjie Tong, Kun Chen, Jiali Yao

**Affiliations:** 1 Department of Critical Care Medicine, Jinhua Hospital Affiliated to Zhejiang University, Jinhua, Zhejiang, China; 2 Department of Stomatology, Affiliated Jinhua Hospital, Zhejiang University School of Medicine, Jinhua Municipal Central Hospital, Jinhua, China

**Keywords:** critical illness, dementia, ischemic stroke, mortality, statin

## Abstract

**Introduction:**

Ischemic stroke is a leading cause of mortality and disability, but the effect of statins on survival in critically ill patients, especially those with dementia, remains unclear.

**Methods:**

This retrospective cohort study analyzed 1,964 patients from MIMIC-IV and validated findings in 4,255 external cases. Time-dependent Cox, IPTW, and PSM were applied.

**Results:**

Statin use was significantly associated with reduced mortality at 30, 90, 180 days, and 1 year, though the effect attenuated over time. A significant statin-dementia interaction was observed, with greater benefit in dementia patients (adjusted HR = 0.548; NNT = 7 vs. 13). The strongest protection occurred immediately post-admission (74.1% risk reduction) and waned by 1 year. Results were robust in sensitivity and external validation.

**Discussion:**

In conclusion, statin therapy improves short-to-medium-term survival in critically ill ischemic stroke patients, with potential additional benefits observed in those with dementia. These findings support early statin use and suggest dementia as a potential effect modifier, though prospective validation with standardized cognitive assessments is needed.

## Introduction

Ischemic stroke represents a paramount global health challenge, constituting approximately 62% of all cerebrovascular events worldwide and ranking as the second leading cause of mortality and third leading cause of disability globally according to the most recent Global Burden of Disease Study (GBD 2021 Nervous System Disorders Collaborators, 2024). The prognosis remains particularly concerning, with contemporary studies demonstrating 5-years survival rates ranging from 45% to 60% depending on stroke severity, age, and comorbidity burden (Tsao et al., 2023). This sobering prognosis is especially evident in severe stroke cases, where 5-years survival rates plummet below 40%, highlighting the critical need for effective therapeutic interventions (Virani et al., 2021). The complex pathophysiology involves not only the initial ischemic insult but also subsequent inflammatory cascades, blood-brain barrier disruption, and secondary neuronal injury that collectively contribute to poor outcomes. Additionally, stroke survivors face substantially elevated risks of cardiovascular complications including myocardial infarction, heart failure, and venous thromboembolism, which collectively contribute to the high long-term mortality rates (GBD 2019 Stroke Collaborators, 2021). The functional outcomes are equally concerning, with approximately half of survivors experiencing moderate to severe disability that significantly compromises quality of life and independence (Ovbiagele et al., 2013).

Statins (3-hydroxy-3-methylglutaryl-coenzyme A reductase inhibitors) have emerged as cornerstone therapies in ischemic stroke management through dual mechanistic pathways (Mach et al., 2020). The primary mechanism involves substantial reduction of low-density lipoprotein cholesterol (LDL-C) by 30%–50% through competitive inhibition of HMG-CoA reductase, facilitating atherosclerotic plaque stabilization and regression (Ray et al., 2020). Beyond lipid modulation, statins demonstrate pleiotropic effects including anti-inflammatory properties through nuclear factor kappa-B inhibition, endothelial function improvement via enhanced nitric oxide bioavailability, and direct neuroprotective effects through modulation of cerebral blood flow and reduction of excitotoxicity (Oesterle et al., 2017). Current guidelines strongly recommend high-intensity statin therapy for secondary prevention in ischemic stroke patients, supported by robust evidence from randomized controlled trials including the SPARCL study, which demonstrated a 16% relative risk reduction in recurrent stroke with atorvastatin 80 mg daily (Kleindorfer et al., 2021).

In severe ischemic stroke management, contemporary evidence presents clinical equipoise (Lee et al., 2022). While the SPARCL trial subanalysis demonstrated that high-dose atorvastatin reduced recurrent stroke risk by 23% in severe stroke patients, contemporary observational studies suggest potential concerns regarding hemorrhagic transformation risk (OR 1.25, 95% CI 1.02–1.53) particularly in patients receiving reperfusion therapies (Amarenco et al., 2020). This therapeutic paradox is further complicated by heterogeneous treatment responses across stroke severity subgroups, with recent investigations exploring optimal timing and intensity of baseline statin use. The 2023 S-STAT randomized trial published in JAMA Neurology found that ultra-early baseline statin use within 24 h was safe but did not significantly improve functional outcomes at 90 days in severe stroke patients, though suggesting potential long-term mortality benefits (Kim et al., 2023).

Accumulating evidence supports the association between statin use and reduced mortality in ischemic stroke. A comprehensive 2022 meta-analysis incorporating 18 observational studies and 3 randomized trials demonstrated that statin therapy was associated with a 22% reduction in all-cause mortality following ischemic stroke (RR 0.78, 95% CI 0.72–0.85) (Park et al., 2025). However, this analysis identified critical evidence gaps regarding temporal patterns of mortality risk reduction and differential effects across patient subgroups defined by stroke severity, etiology, and comorbidities. The relationship appears duration-dependent, with more pronounced benefits observed in long-term versus short-term mortality, as demonstrated in a recent prospective cohort study showing 35% reduction in 3-years mortality versus 18% reduction in 30-days mortality associated with pre-stroke statin use (Sabatine et al., 2017).

This study aims to provide valuable insights into the management of ischemic stroke in critically ill populations through an analysis of patient demographics, treatment protocols, and clinical outcomes. The primary objective is to evaluate the survival benefits associated with statin use and to examine how these benefits may vary in the presence of comorbid dementia. Ultimately, the research seeks to enhance clinical decision-making and patient care in critical settings. The findings are expected to lay the groundwork for future research and inform clinical practice, with the goal of reducing the burden of ischemic stroke and improving outcomes for affected individuals.

## Materials and methods

This study adopted a retrospective cohort study design, analyzing data based on the MIMIC-IV database (Medical Information Mart for Intensive Care IV, version 3.1). The MIMIC-IV database contains patient information from the Emergency Department and Intensive Care Unit (ICU) of the Beth Israel Deaconess Medical Center in Boston, Massachusetts, USA, encompassing information on over 65,000 ICU patients and more than 200,000 emergency department visits. The author (Jiali Yao) was certified through the Collaborative Institutional Training Initiative (CITI) program and obtained data access (certification number: 68028865). To ensure patient privacy, all data in MIMIC-IV have been de-identified, thus informed consent was not required. Data of ICU patients from Jinhua Hospital in Zhejiang Province between 2010 and 2025 were additionally collected for external validation. This study was approved by the Ethics Committee of Jinhua Hospital of Zhejiang Province (Approval No.: 2025-188).

### Study subjects

We used Navicat Premium to filter all adult patients (≥18 years old) who were admitted to the ICU for the first time with a primary diagnosis of ischemic stroke (based on diagnostic codes in the MIMIC-IV database, including both ICD-9 and ICD-10 versions) (*n* = 5279) (Figure 1). Exclusion criteria included: (1) patients with multiple ICU admissions; (2) patients with an ICU stay of less than 24 h; (3) patients with missing critical clinical data, defined as missing data on the primary outcome (all-cause mortality), disease severity scores (GCS, SOFA, OASIS, SAPSII), or essential laboratory parameters and covariates (including age, sex, race, and key comorbidities) required for multivariable model adjustment. To verify the core findings of this study, external validation was further performed using real-world clinical ICU data from Jinhua Hospital, Zhejiang Province, with the same inclusion and exclusion criteria adopted (*n* = 4255).

**FIGURE 1:**
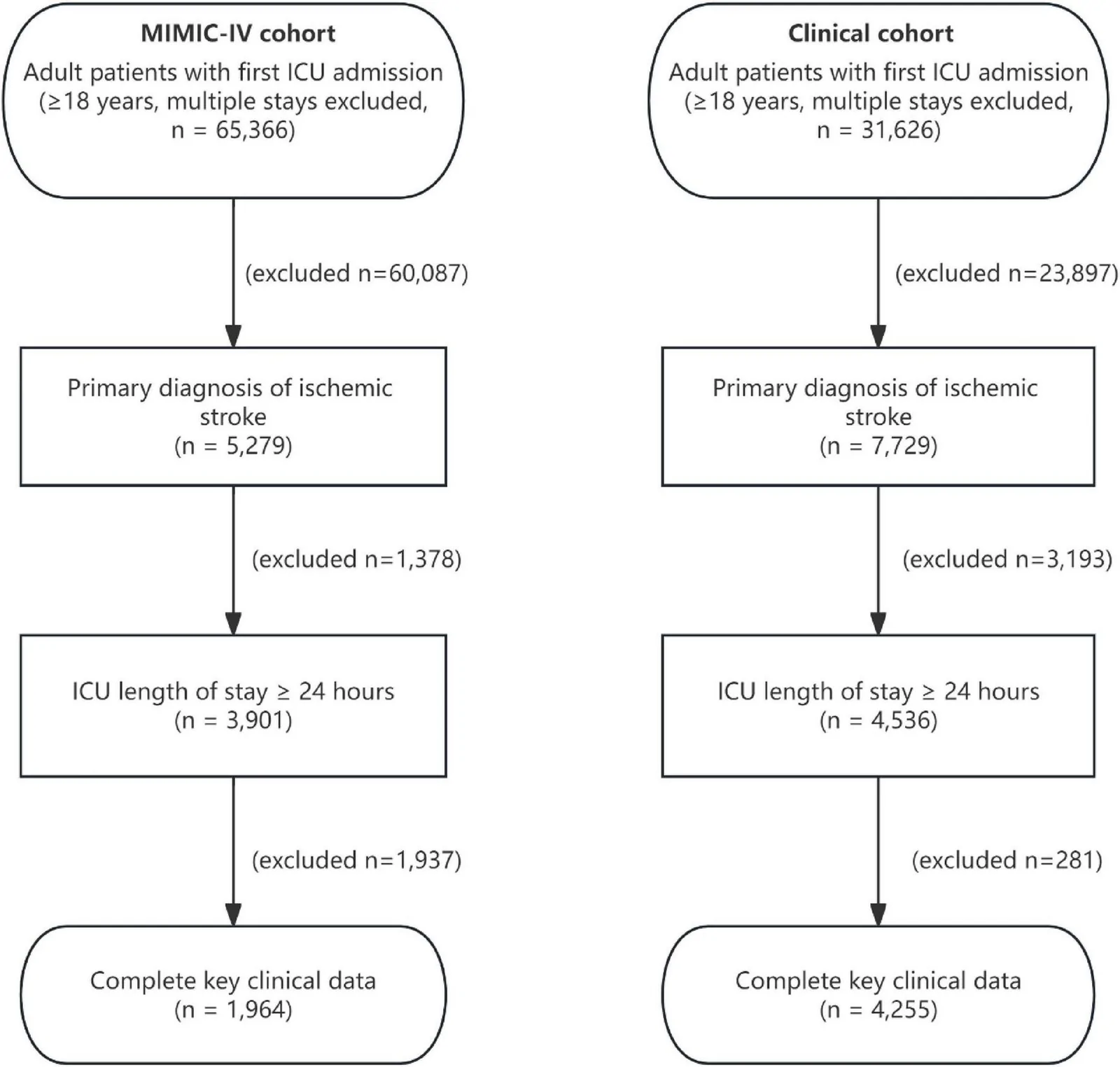
Flowchart of patient selection for the MIMIC-IV derivation cohort and the external validation cohort. Flowchart illustrating the stepwise patient selection process for the derivation cohort (MIMIC-IV database, *n* = 1,964) and the external validation cohort (Jinhua Hospital, *n* = 4,255). Numbers in parentheses indicate patients excluded at each stage.

### Data collection

Demographic characteristics were collected, including age, sex, race, height, weight, and calculated BMI. Clinical biochemical test results were collected (anion gap, bicarbonate, chloride, sodium, potassium, creatinine, glucose, international normalized ratio, prothrombin time, thrombin time, hemoglobin, mean corpuscular hemoglobin, mean corpuscular hemoglobin concentration, mean corpuscular volume, red blood cells, red cell distribution width, white blood cells, platelets) along with disease severity scores. These scoring systems included SOFA, SAPS II, OASIS, SIRS and GCS. Among them, APACHE-IV and GCS were predominantly utilized in the clinical cohort. The Charlson Comorbidity Index and various comorbidities were also collected, including hypertension, diabetes, dementia, kidney disease, myocardial infarction, congestive heart failure, cerebrovascular disease, peripheral vascular disease, chronic lung disease, rheumatic disease, peptic ulcer disease, mild liver disease, paraplegia, malignancy, severe liver disease, and metastatic solid tumors. Covariates were selected *a priori* based on clinical plausibility and prior literature (Fan et al., 2025; Luo et al., 2026; Zhang et al., 2026), as these variables are established prognostic factors in critically ill stroke patients and potential confounders of the association between statin use and mortality.

In the external validation cohort from Jinhua hospital, dementia was identified using clinically documented diagnoses that required: (1) acquired and progressive brain functional impairment; (2) impairment in at least two cognitive domains assessed in the conscious state; (3) cognitive decline significantly interfering with daily living and social functioning; and (4) explicit exclusion of delirium, psychiatric disorders, and metabolic disturbances as alternative causes. This diagnostic approach aligns with internationally recognized criteria (e.g., DSM-5, ICD-11). It is important to acknowledge that in the MIMIC-IV cohort, ICD-based coding in critically ill stroke patients may not fully distinguish pre-existing dementia from acute encephalopathy, delirium, or post-stroke cognitive impairment; this limitation is addressed in the discussion.

The primary exposure variable was the use of statins, which included all types such as atorvastatin, rosuvastatin, simvastatin, pravastatin, lovastatin, fluvastatin, and pitavastatin. Statin use was defined as documented prescription at the time of ICU admission, ascertained from the patient’s home medication list or admission medication reconciliation. Thus, the exposure reflects baseline statin use (i.e., pre-admission or ongoing therapy) rather than *de novo* initiation during the ICU stay.

The primary outcome was all-cause mortality, assessed at four time points: 30-days, 90-days, 180-days, and 1-year mortality. Survival time was calculated from ICU admission until death or censoring. Due to the lack of long-term follow-up data in the clinical cohort, only the 30-days all-cause mortality recorded during hospitalization was evaluated. In both the MIMIC-IV and external validation cohorts, this outcome was calculated as the proportion of patients with ischemic stroke who died during follow-up, which aligns with the epidemiologic concept of case-fatality. To reflect both the all-cause nature of the outcome and the disease-specific denominator, we refer to this measure as all-cause case-fatality throughout the manuscript (Laupland et al., 2021, 2022).

### Data analysis

All analyses were performed using R software (version 4.3.1). Variables with >20% missing data were excluded (Luo et al., 2023); those with <5% missingness were imputed using multiple imputation. Outliers were winsorized at the 1st and 99th percentiles. Baseline characteristics were compared using appropriate statistical tests, and survival differences were assessed via Kaplan-Meier curves with log-rank tests. The proportional hazards (PH) assumption was evaluated using Schoenfeld residuals. Since statin use violated the PH assumption at all time points, we employed time-dependent Cox models with a statin-by-log(time) interaction term; model fit was compared using Akaike Information Criterion (AIC). For multivariable analysis, we used a stratified Cox model for the 30-days endpoint (which satisfied the PH assumption after stratification by race) and time-dependent Cox models for longer endpoints, adjusting for age, sex, and race. Subgroup analyses were performed by age, sex, race, and six major comorbidities (diabetes, hypertension, kidney disease, heart failure, cerebrovascular disease, and dementia), with interaction terms introduced to test effect modification.

To address baseline imbalances, we applied inverse probability of treatment weighting (IPTW) using propensity scores, with extreme weights trimmed at the 99th percentile, and assessed balance using standardized mean differences (SMD < 0.1 considered negligible). Propensity score matching (PSM) was performed using nearest-neighbor matching at a 1:2 ratio. Model performance was evaluated using the concordance index and Bayesian Information Criterion, with likelihood ratio tests for nested model comparisons. The number needed to treat was calculated as 1/absolute risk reduction. External validation was performed using the Jinhua Hospital cohort with univariate and multivariable Cox regression models, adjusting for age, sex, and ethnicity, and including the statin-by-dementia interaction term. Statistical significance was defined as two-tailed *P* < 0.05, and all effect estimates are reported as hazard ratios with 95% confidence intervals.

## Results

Baseline characteristics of the 1,964 included patients, stratified by statin use, are presented in Table 1. Patients receiving statins were significantly older, had a higher proportion of males, and had lower acute disease severity scores (OASIS, SIRS, SOFA) but higher GCS scores compared to non-users (all *P* < 0.05). As expected, the statin group exhibited a higher burden of chronic comorbidities, including renal disease, myocardial infarction, congestive heart failure, and diabetes (all *P* < 0.01), and consequently a higher Charlson Comorbidity Index (6.41 ± 2.66 vs. 5.32 ± 2.82, *P* < 0.001). Detailed between-group comparisons are provided in Table 1.

**TABLE 1:** Baseline information table for severe ischemic stroke patients regarding statin use.

Variable	Level	Overall	No statin	Statin	*P*-value
		(*N* = 1964)	(*N* = 611)	(*N* = 1353)	
Age [mean (SD)]		68.27 (14.78)	64.13 (17.33)	70.14 (13.05)	<0.001
Gender (%)	F	891 (45.4)	298 (48.8)	593 (43.8)	0.047
	M	1073 (54.6)	313 (51.2)	760 (56.2)	
Race (%)	White	1161 (59.1)	351 (57.4)	810 (59.9)	<0.001
	Black	229 (11.7)	49 (8.0)	180 (13.3)	
	Other	574 (29.2)	211 (34.5)	363 (26.8)	
Weight [mean (SD)]		80.67 (21.30)	79.52 (22.55)	81.19 (20.70)	0.107
Height [mean (SD)]		168.68 (10.82)	168.59 (11.11)	168.72 (10.69)	0.805
BMI [mean (SD)]		28.25 (6.67)	27.83 (6.94)	28.43 (6.55)	0.063
Aniongap [mean (SD)]		14.39 (4.20)	14.73 (4.28)	14.24 (4.16)	0.019
Bicarbonate [mean (SD)]		22.53 (3.97)	22.13 (4.12)	22.71 (3.89)	0.003
Chloride [mean (SD)]		104.63 (6.05)	104.81 (6.28)	104.54 (5.94)	0.358
Sodium [mean (SD)]		139.09 (4.94)	139.07 (5.35)	139.10 (4.75)	0.893
Potassium [mean (SD)]		4.16 (0.71)	4.13 (0.73)	4.17 (0.69)	0.272
Creatinine [mean (SD)]		1.32 (1.14)	1.31 (1.14)	1.33 (1.14)	0.746
Glucose [mean (SD)]		152.33 (66.81)	148.60 (61.42)	154.01 (69.06)	0.096
INR [mean (SD)]		1.40 (0.64)	1.44 (0.86)	1.37 (0.51)	0.031
PT [mean (SD)]		15.14 (5.16)	15.43 (5.45)	15.01 (5.01)	0.091
PTT [mean (SD)]		36.87 (23.06)	35.53 (20.73)	37.48 (24.03)	0.083
Hemoglobin [mean (SD)]		11.23 (2.43)	11.21 (2.32)	11.23 (2.48)	0.848
MCH [mean (SD)]		29.94 (2.64)	30.10 (2.61)	29.86 (2.65)	0.058
MCHC [mean (SD)]		32.87 (1.59)	32.90 (1.64)	32.85 (1.57)	0.579
MCV [mean (SD)]		91.11 (6.81)	91.58 (6.98)	90.90 (6.73)	0.04
RBC [mean (SD)]		3.76 (0.81)	3.74 (0.79)	3.78 (0.82)	0.307
RDW [mean (SD)]		14.59 (2.02)	14.69 (2.09)	14.54 (1.99)	0.131
WBC [mean (SD)]		11.89 (5.72)	12.43 (6.54)	11.65 (5.29)	0.005
Platelet [mean (SD)]		209.14 (96.89)	206.57 (105.45)	210.30 (92.79)	0.43
OASIS [mean (SD)]		34.06 (8.82)	34.81 (9.26)	33.72 (8.59)	0.011
SAPSII [mean (SD)]		38.00 (13.79)	38.63 (15.15)	37.71 (13.13)	0.173
SIRS [mean (SD)]		2.57 (0.98)	2.76 (0.92)	2.49 (1.00)	<0.001
SOFA [mean (SD)]		4.95 (3.47)	5.35 (3.90)	4.77 (3.25)	0.001
GCS [mean (SD)]		12.88 (3.35)	12.59 (3.68)	13.01 (3.18)	0.009
Renal disease (%)	0	1558 (79.3)	531 (86.9)	1027 (75.9)	<0.001
	1	406 (20.7)	80 (13.1)	326 (24.1)	
Myocardial infarct (%)	0	1563 (79.6)	547 (89.5)	1016 (75.1)	<0.001
	1	401 (20.4)	64 (10.5)	337 (24.9)	
Congestive heart failure (%)	0	1396 (71.1)	479 (78.4)	917 (67.8)	<0.001
	1	568 (28.9)	132 (21.6)	436 (32.2)	
Cerebrovascular disease (%)	0	405 (20.6)	123 (20.1)	282 (20.8)	0.764
	1	1559 (79.4)	488 (79.9)	1071 (79.2)	
Peripheral vascular disease (%)	0	1646 (83.8)	534 (87.4)	1112 (82.2)	0.005
	1	318 (16.2)	77 (12.6)	241 (17.8)	
Dementia (%)	0	1872 (95.3)	582 (95.3)	1290 (95.3)	1
	1	92 (4.7)	29 (4.7)	63 (4.7)	
Chronic pulmonary disease (%)	0	1571 (80.0)	501 (82.0)	1070 (79.1)	0.152
	1	393 (20.0)	110 (18.0)	283 (20.9)	
Rheumatic disease (%)	0	1905 (97.0)	590 (96.6)	1315 (97.2)	0.54
	1	59 (3.0)	21 (3.4)	38 (2.8)	
Peptic ulcer disease (%)	0	1919 (97.7)	604 (98.9)	1315 (97.2)	0.034
	1	45 (2.3)	7 (1.1)	38 (2.8)	
Mild liver disease (%)	0	1824 (92.9)	527 (86.3)	1297 (95.9)	<0.001
	1	140 (7.1)	84 (13.7)	56 (4.1)	
Diabetes (%)	0	1306 (66.5)	489 (80.0)	817 (60.4)	<0.001
	1	658 (33.5)	122 (20.0)	536 (39.6)	
Paraplegia (%)	0	1305 (66.4)	434 (71.0)	871 (64.4)	0.005
	1	659 (33.6)	177 (29.0)	482 (35.6)	
Malignant cancer (%)	0	1797 (91.5)	541 (88.5)	1256 (92.8)	0.002
	1	167 (8.5)	70 (11.5)	97 (7.2)	
Severe liver disease (%)	0	1911 (97.3)	578 (94.6)	1333 (98.5)	<0.001
	1	53 (2.7)	33 (5.4)	20 (1.5)	
Metastatic solid tumor (%)	0	1903 (96.9)	586 (95.9)	1317 (97.3)	0.121
	1	61 (3.1)	25 (4.1)	36 (2.7)	
Charlson Comorbidity Index [mean (SD)]		6.07 (2.76)	5.32 (2.82)	6.41 (2.66)	<0.001
Hypertension (%)	0	705 (35.9)	234 (38.3)	471 (34.8)	0.15
	1	1259 (64.1)	377 (61.7)	882 (65.2)	
Ventilation (%)	0	1506 (76.7)	448 (73.3)	1058 (78.2)	0.021
	1	458 (23.3)	163 (26.7)	295 (21.8)	
Age score [mean (SD)]		2.34 (1.30)	2.01 (1.41)	2.49 (1.21)	<0.001

BMI, body mass index; INR, international normalized ratio; PT, prothrombin time; PTT, part prothrombin time; MCH, mean corpuscular hemoglobin; MCHC, mean corpuscular hemoglobin concentration; MCV, erythrocyte mean corpuscular volume; RBC, red blood cell; RDW, red blood cell distribution width; WBC, white blood cell; OASIS, oxford acute severity of illness score; SAPSII, simplified acute physiology score ii; SIRS, systemic inflammatory response syndrome; SOFA, sequential organ failure assessment; GCS, Glasgow coma scale.

Significant differences in baseline comorbidities and statin utilization rates were observed between the external validation clinical cohort and the primary MIMIC-IV cohort (SMD > 0.1, Supplementary Table 1), indicating population heterogeneity across different data sources. Despite these baseline discrepancies, these variables were adjusted in the multivariate regression analysis to mitigate confounding bias and verify the robustness of the primary effects.

Patients in the statin treatment group exhibited longer survival times compared with those in the non-statin group, although the difference did not reach statistical significance (*P* = 0.074). However, significant between-group differences in survival times were observed at 30 days, 90 days, 180 days, and 1 year (Table 2). Mortality rates were consistently lower in the statin group than in the non-statin group at 30 days (16.8% vs. 33.4%), 90 days (23.4% vs. 38.3%), 180 days (26.9% vs. 39.9%), and 1 year (30.8% vs. 40.7%). Kaplan–Meier survival analysis revealed significant differences between the two groups at all time points, with the statin treatment group demonstrating a significantly better prognosis than the non-statin group (Figure 2).

**TABLE 2:** Survival status and length of stay (LOS) of severe stroke patients stratified by statin therapy at various time points.

Variable	Level	Overall	No statin	Statin	*P*-value
		(*N* = 1964)	(*N* = 611)	(*N* = 1353)	
Survival time [mean (SD)]		212.22 (620.01)	175.04 (585.51)	229.01 (634.47)	0.074
30-days mortality status (%)	0	1533 (78.1)	407 (66.6)	1126 (83.2)	<0.001
	1	431 (21.9)	204 (33.4)	227 (16.8)	
los_30d [mean (SD)]		17.02 (10.47)	15.82 (10.20)	17.56 (10.56)	0.001
90-days mortality status (%)	0	1414 (72.0)	377 (61.7)	1037 (76.6)	<0.001
	1	550 (28.0)	234 (38.3)	316 (23.4)	
los_90d [mean (SD)]		30.60 (32.06)	26.94 (30.21)	32.25 (32.73)	0.001
180-days mortality status (%)	0	1356 (69.0)	367 (60.1)	989 (73.1)	<0.001
	1	608 (31.0)	244 (39.9)	364 (26.9)	
los_180d [mean (SD)]		45.88 (62.79)	39.86 (59.28)	48.59 (64.14)	0.004
1-y mortality status (%)	0	1274 (64.9)	338 (55.3)	936 (69.2)	<0.001
	1	690 (35.1)	273 (44.7)	417 (30.8)	
los_1y [mean (SD)]		69.79 (118.31)	59.73 (110.28)	74.33 (121.52)	0.011

LOS, length of stay; SD, standard deviation. Survival status is presented as “0” (alive) and “1” (deceased). Data for survival status are presented as counts (percentages), and LOS data are presented as mean (SD). *P*-values were derived from the Chi-square test (for status) and Student’s *t*-test (for LOS). All analyses were conducted based on the total cohort of 1,964 patients.

**FIGURE 2:**
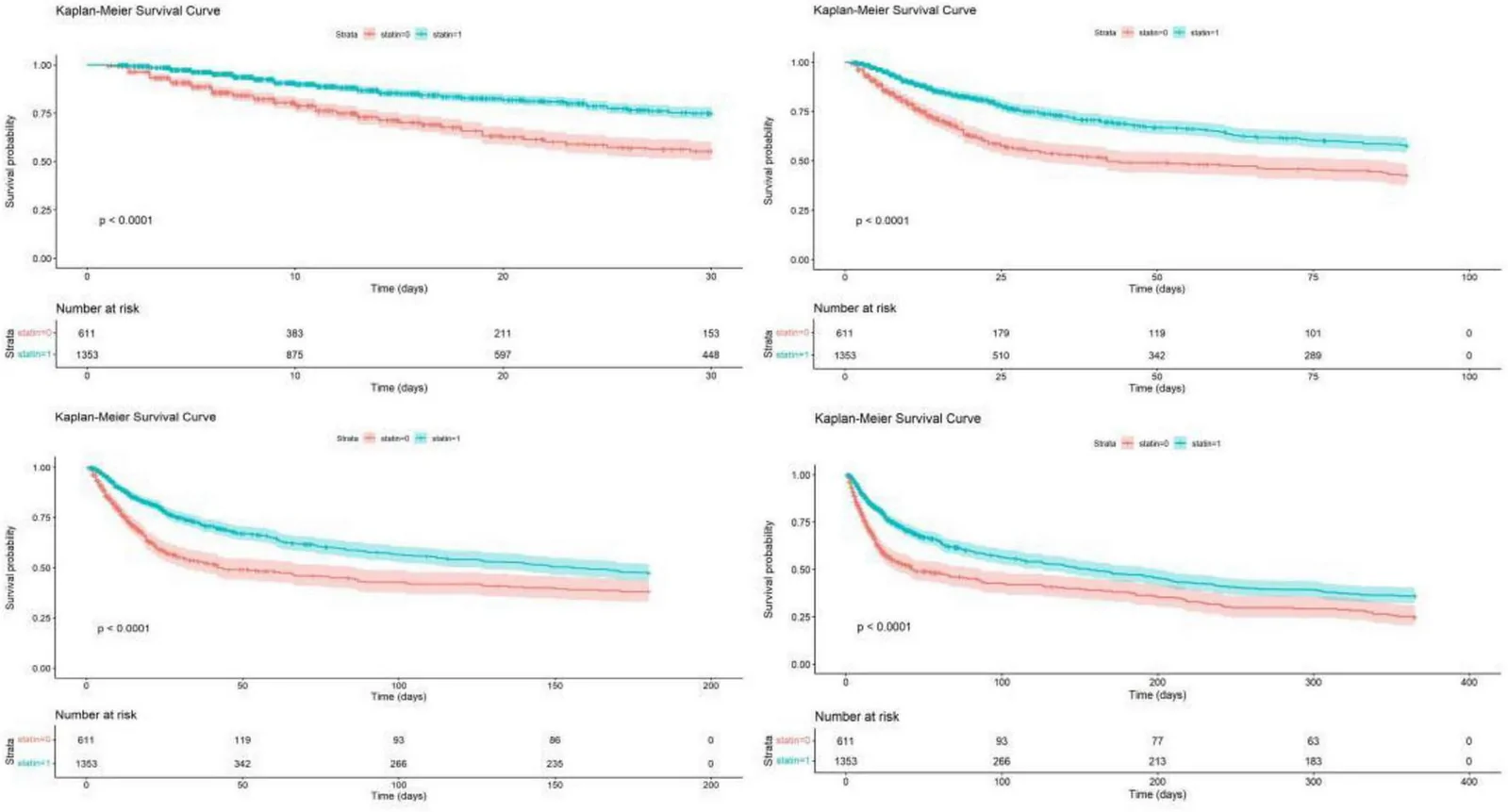
Survival curves of severe ischemic stroke patients with and without statin use at different time points. At 30 days, 90 days, 180 days, and 1 year, the *P*-values were all >0.001, indicating that patients ed with statins had longer survival than those not treated with statins.

Univariate Cox regression (Table 3) showed that statin use was significantly associated with reduced mortality at all time points, with HRs ranging from 0.455 (30-days) to 0.589 (1-year), all *P* < 0.001. The proportional hazards assumption was violated for statin use at all time points (all *P* < 0.05). A time-dependent Cox model demonstrated significantly better fit than the traditional model (all *P* < 0.05, Table 4), revealing a time-varying protective pattern: the protective effect of statins was strongest immediately post-admission (HR = 0.164–0.251) and gradually attenuated over time (Table 5).

**TABLE 3:** Univariate Cox regression analysis of the association between statin therapy and mortality at different time points.

Group	Time	HR	95% CI	*P*-value
Statin				
0		–	–	
1	30 days	0.455	0.377–0.550	<0.001
	90 days	0.532	0.449–0.630	<0.001
	180 days	0.582	0.495–0.685	<0.001
	1 year	0.589	0.505–0.686	<0.001

HR, hazard ratio; CI, confidence interval. Reference group for statin therapy is “0” (no statin use). All models are unadjusted. Statistically significant results are indicated by *p* < 0.05.

**TABLE 4:** Comparison of model performance between the traditional Cox regression and the time-dependent Cox model.

Time point	Traditional AIC	Time-dependent AIC	AIC improvement	Concordance	*P*-value
30-days	6059.4	6055	4.5	0.601	0.011
90-days	7533	7516.1	16.9	0.592	<0.001
180-days	8217.1	8187.5	29.6	0.592	<0.001
1-year	9126.7	9108.3	18.4	0.584	<0.001

AIC, Akaike information criterion; C-index, Harrell’s concordance index. The time-dependent Cox model demonstrates superior fit compared to the traditional model, as evidenced by lower AIC values across all time points (all *p* < 0.05). Improvement in AIC was calculated as ΔAIC = AIC *_<suprm>traditional</suprm>_*−AIC *_<suprm>time–dependent</suprm>_*. The likelihood ratio test was used to compare the model fits.

**TABLE 5:** Dynamic protection effect of statin therapy over time based on the time-dependent Cox model.

Time point	Initial HR	Initial protection	Time-dependent HR	Final HR	Final protection
30-days	0.250	75.0%	1.451	0.693	30.7%
90-days	0.173	82.7%	1.532	0.542	45.8%
180-days	0.164	83.6%	1.571	0.512	48.8%
1-year	0.251	74.9%	1.314	0.572	42.8%

HR, hazard ratio; Protection, calculated as (1 − HR). “Initial HR” denotes the protective effect immediately after ICU admission (Day 1). “Final HR” represents the time-weighted average or terminal risk estimate at the specified time point. “Initial protection” and “final protection” represent the corresponding percentage risk reduction. The time-dependent HR coefficient reflects the dynamic hazard across the observation period.

Multivariable Cox regression adjusting for age, sex, and race (Supplementary Table 2) confirmed that statin use was independently associated with reduced mortality at all time points (all *P* < 0.001). Due to PH assumption violations for statin and race variables at longer follow-up, we employed a stratified Cox model for the 30-days endpoint (HR = 0.406, Table 6) and time-dependent Cox models for subsequent endpoints, which confirmed the attenuation of the statin protective effect over time (Figure 3).

**TABLE 6:** Summary of statin protective effects and dynamic attenuation in severe stroke patients.

Time point	Model type	Initial protection	Effect attenuation
30-days	Stratified Cox	59.4% risk reduction	Stable
90-days	Time-dependent	83.7% risk reduction	Observed (HR = 1.51)
180-days	Time-dependent	84.8% risk reduction	Observed (HR = 1.557)
1-year	Time-dependent	77.1% risk reduction	Observed (HR = 1.313)

HR, hazard ratio. “Initial protection” reflects the magnitude of risk reduction (1 − HR) at the onset of statin therapy. “Effect attenuation” indicates the observed decline in protective efficacy over time, quantified by the time-varying HR. Stratified Cox models were utilized for the 30-days endpoint where proportional hazards were assumed, while time-dependent Cox models were employed for subsequent time points to account for non-proportionality.

**FIGURE 3:**
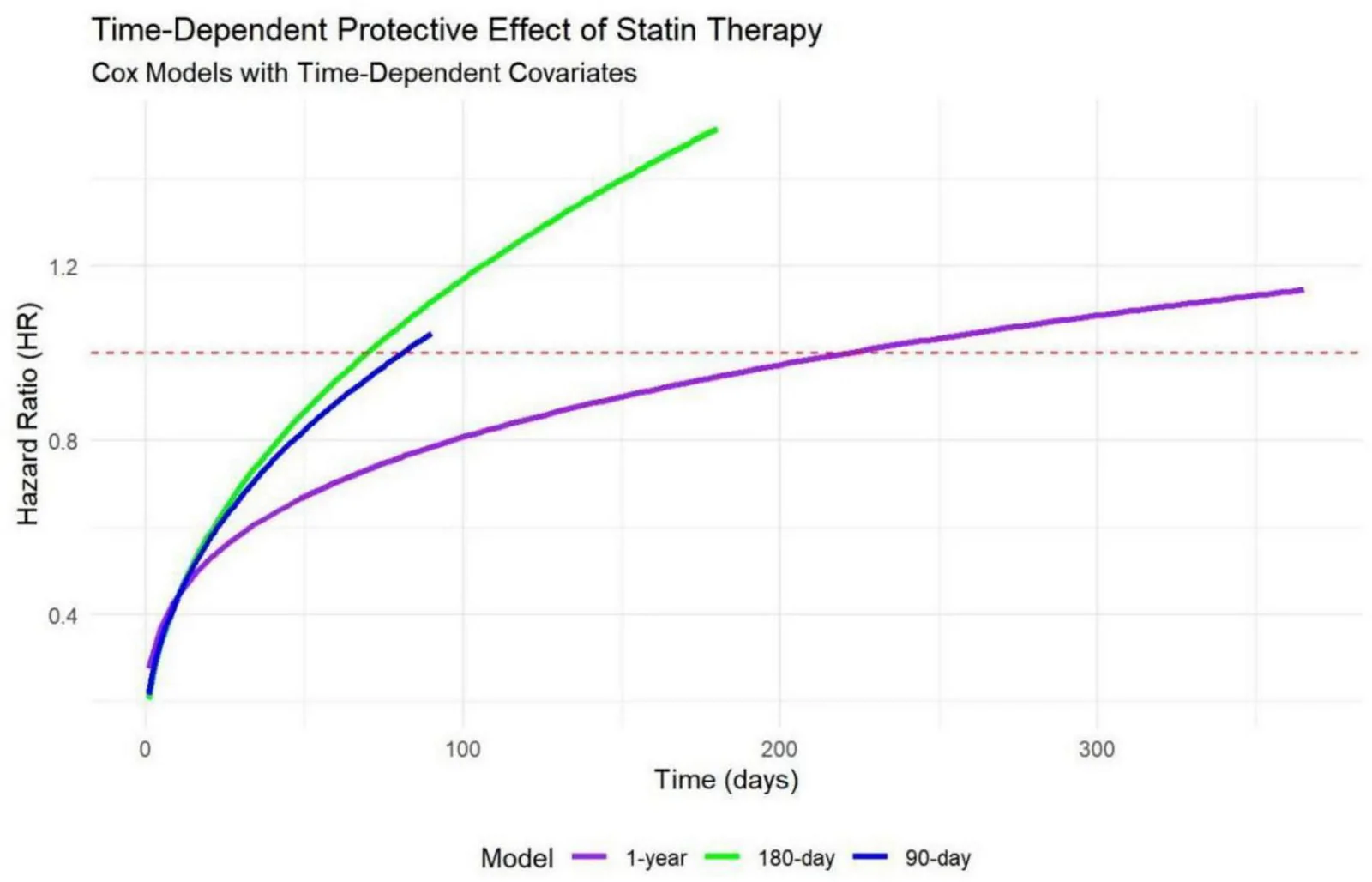
Time-dependent protective effect of statin treatment. The figure shows that the protective effect of statins diminishes over time.

In the external validation cohort, the association between statin use and reduced 30-days mortality was consistently confirmed. Both univariate Cox analysis (HR = 0.622, 95% CI: 0.476–0.811, *p* < 0.001) and multivariate adjustment (adjusted for age, sex and ethnicity) (HR = 0.622, 95% CI: 0.476–0.811, *p* < 0.001) yielded results consistent with those derived from the MIMIC-IV cohort (Supplementary Table 3). Furthermore, the proportional hazards assumption was satisfied. These findings collectively validate the conclusion that statin use reduces short-term mortality in critically ill patients with stroke.

Subgroup analyses (Figure 4 and Supplementary Table 4) showed consistent protective effects of statins across most subgroups. While most subgroups exhibited gradual attenuation of protection over time (average risk reduction declining from 59.5% to 46.4%) (Figure 5), patients with dementia demonstrated the strongest sustained protective effect, with HRs ranging from 0.261 to 0.290 across all time points. Interaction tests (Supplementary Table 5) revealed no significant interactions between statin use and age, sex, race, or most comorbidities. Notably, the statin-dementia interaction became significant from 90 days through 1 year (*P* < 0.05), suggesting additional and sustained protection in this subgroup.

**FIGURE 4:**
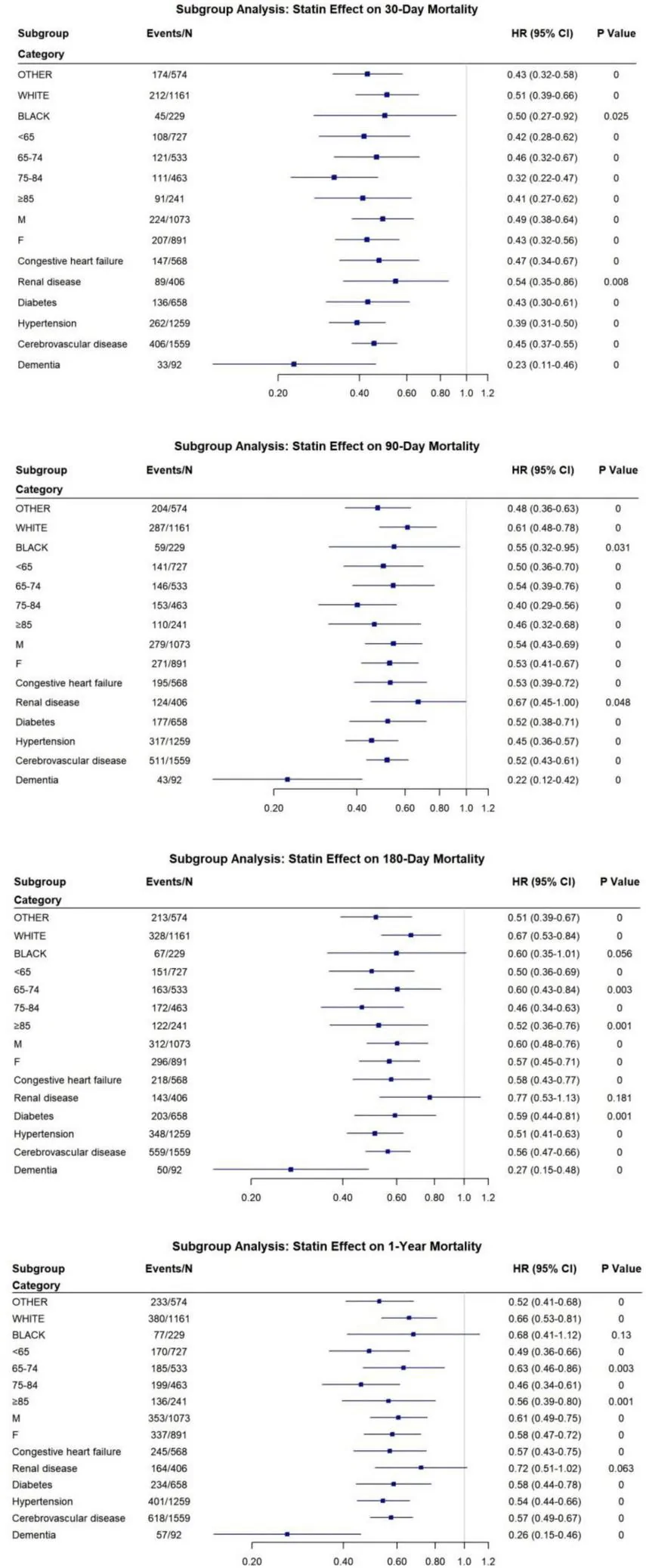
Subgroup analysis: the effect of statin on mortality at different time points. Forest plot showing hazard ratios (HRs) with 95% confidence intervals (CIs) for the effect of statin therapy on mortality of different time points across predefined subgroups. The size of each square is proportional to the subgroup sample size. Horizontal lines represent the 95% CI for each HR. The vertical dashed line at HR = 1.0 indicates no effect. The *P*-values for interaction are displayed on the right and were derived from multivariable Cox regression models including an interaction term between statin use and the subgroup variable, adjusted for age, sex and major comorbidities.

**FIGURE 5:**
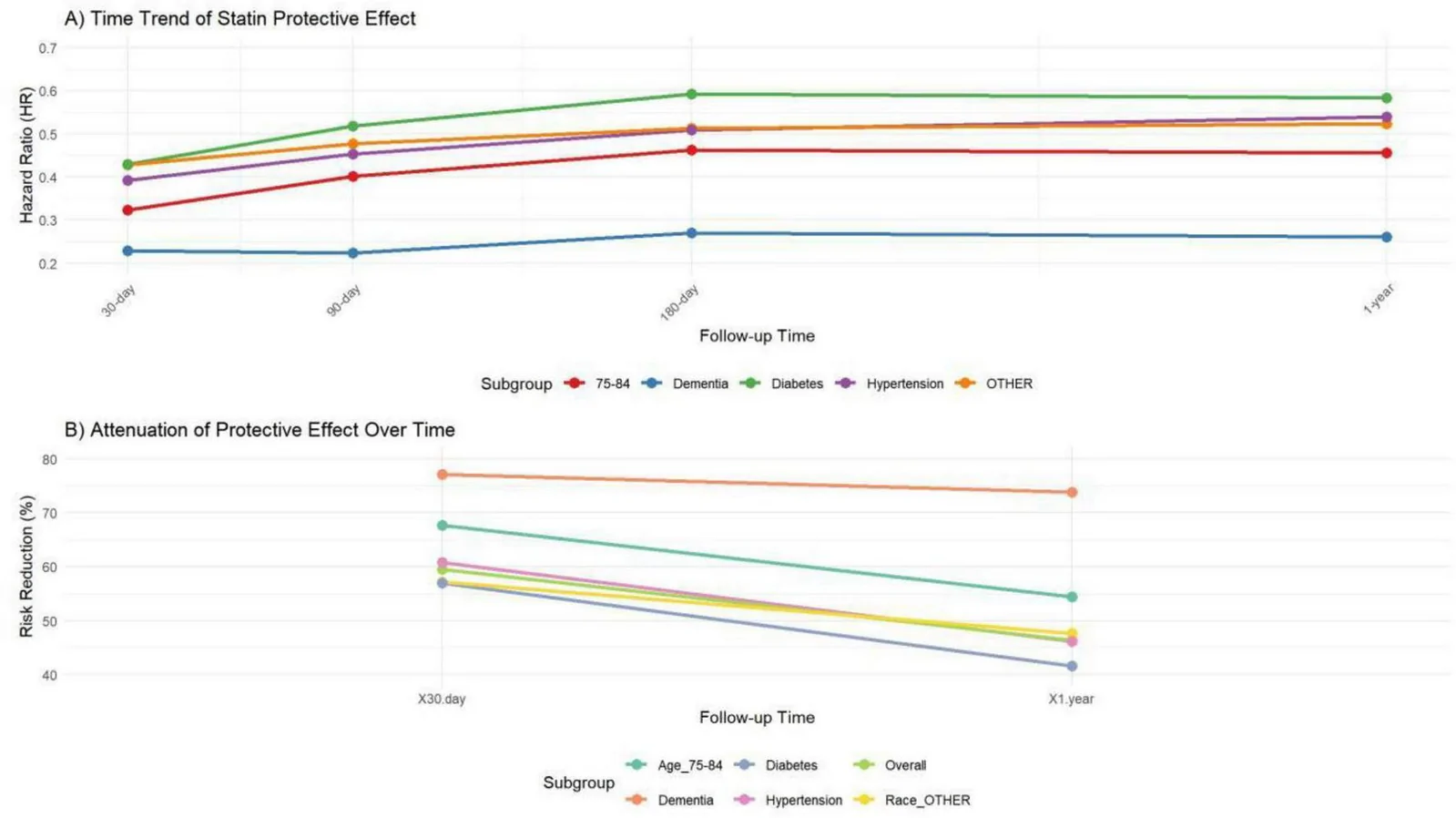
Time trends of statin protection effect **(A)** and the attenuation of protective effect over time **(B)**. The figure shows that the protective effect of statins on various subgroups attenuates and decays over time.

Among the 92 patients (4.7%) with dementia (Supplementary Table 6), significant baseline differences were observed compared to non-dementia patients, including older age, higher disease severity, and greater diabetes prevalence. IPTW successfully balanced these covariates (all SMD < 0.1, Supplementary Figure 1). In the multivariable-adjusted Cox model, the statin-dementia interaction was significant (HR = 0.548, 95% CI: 0.314–0.955, *P* = 0.034), indicating a 45.2% mortality risk reduction associated with statins in dementia patients. This finding was supported by IPTW analysis (HR = 0.483, 95% CI: 0.267–0.876, *P* = 0.017), while PSM showed a consistent trend despite reduced statistical power (HR = 0.601, *P* = 0.147). The likelihood ratio test supported the interaction model (*P* = 0.037), with good model discrimination (*C* = 0.672).

Time-dependent Cox modeling confirmed the attenuation of the statin protective effect over time (time-dependent HR = 1.296, *P* < 0.001). The consistency of effect direction across multiple analytical approaches is presented in Supplementary Figure 2. The strongest protection was observed immediately post-admission (74.1% risk reduction on day 1, Supplementary Figure 3), which gradually diminished by day 365 (HR = 1.197). The statin-dementia interaction remained marginally significant in this time-dependent model (HR = 0.582, 95% CI: 0.334–1.016, *P* = 0.057). Survival curves (Figure 6) showed that dementia patients not receiving statins had the worst outcomes, while statin-treated dementia patients had outcomes superior to non-dementia non-users. The number needed to treat for dementia patients was 7 (ARR = 15%), compared to 13 (ARR = 8%) for non-dementia patients (Supplementary Figure 4), suggesting greater clinical benefit in the dementia subgroup.

**FIGURE 6:**
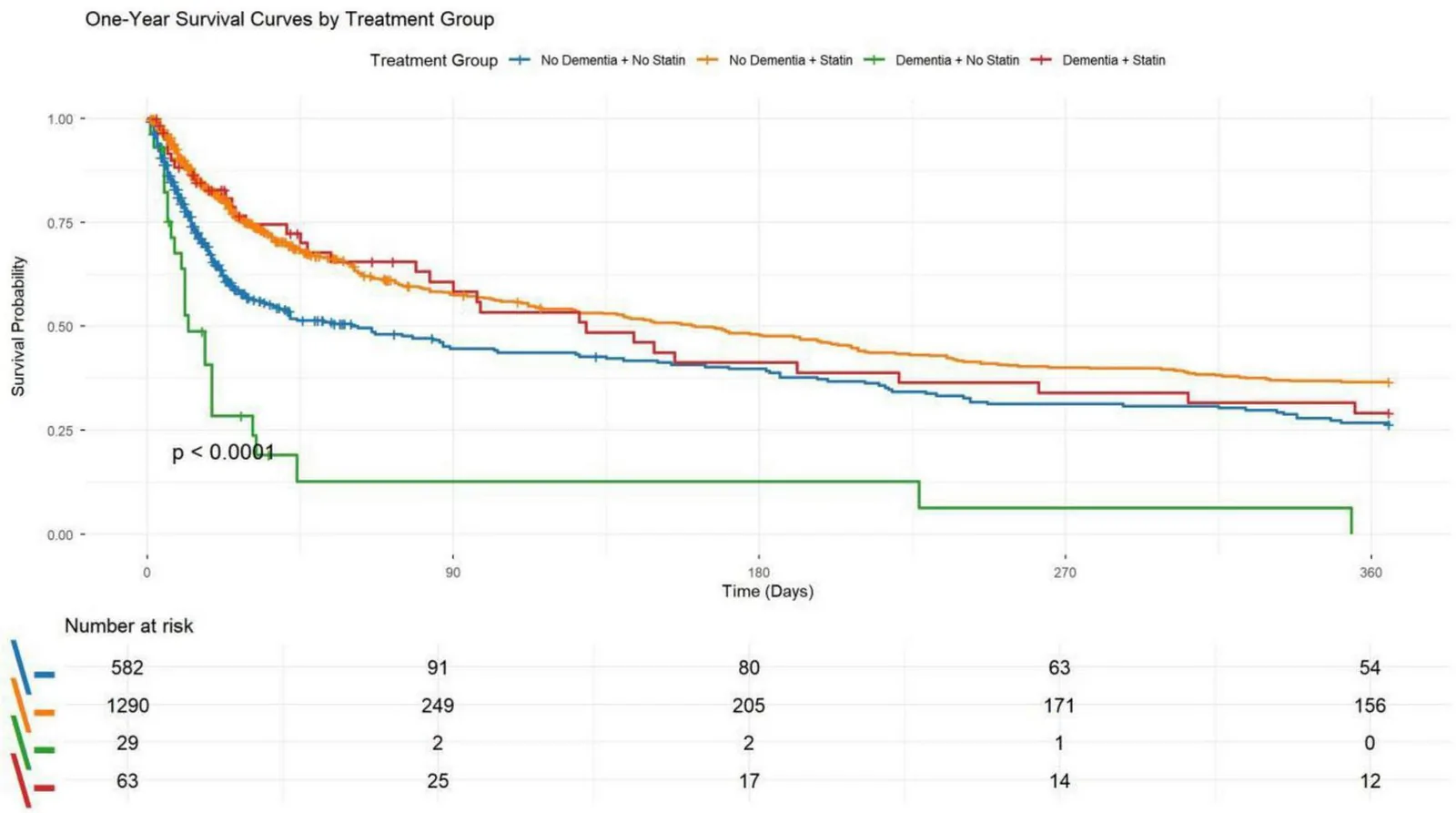
Effect of statin treatment on survival curves in patients with and without dementia. Patients with dementia who did not receive statin treatment had the worst survival outcomes, while those with dementia who received statin treatment had outcomes second only to those with dementia who received statin treatment.

In the external validation cohort, although the interaction failed to reach statistical significance (Supplementary Table 3), the effect direction of statin therapy in the dementia subgroup remained consistent with that in the primary cohort (Supplementary Figure 5). These findings support the plausibility of this effect modification, while its robustness needs to be further verified in larger sample cohorts.

## Discussion

Despite advances in acute stroke management, optimal therapeutic strategies for critically ill ischemic stroke patients remain an area of active investigation (Wang et al., 2019). Previous research has suggested that statins may exert neuroprotective effects, thereby enhancing survival outcomes in stroke patients (Rieke et al., 1991). By analyzing data from critically ill patients with ischemic stroke sourced from the MIMIC-IV database, the objective is to evaluate the association between statin use and all-cause mortality at multiple time points. The findings from this research may have significant implications on clinical practices, highlighting the need for timely and effective management strategies to improve patient outcomes in this vulnerable population (Chen et al., 2019).

Baseline analysis revealed that statin users were older, had more chronic comorbidities, and a higher Charlson Comorbidity Index, reflecting real-world prescribing patterns where clinicians preferentially prescribe statins for secondary prevention in high-risk patients (O’Brien and Greene, 2019). Notably, although patients in the statin group had a heavier burden of chronic comorbidities, their acute disease severity scores (OASIS, SAPSII, SOFA) were lower, while GCS scores were higher, suggesting potential neuroprotective effects of statins during the acute phase of severe ischemic stroke (Huang et al., 2024). Laboratory findings further supported this interpretation, with the statin group showing lower anion gap, higher bicarbonate levels, and lower white blood cell count and INR, consistent with the known anti-inflammatory properties of statins (Ayyalu et al., 2025).

Our findings demonstrate that baseline statin use is consistently associated with reduced mortality at all time points from 30 days to 1 year, suggesting that the protective effects of ongoing statin therapy may extend beyond the acute phase. Unlike previous studies that primarily focused on short-term outcomes or animal models (Sveinsson et al., 2014), our findings indicate that statin use is consistently associated with reduced all-cause mortality at various time points post-stroke. However, it is important to note that our exposure reflects pre-admission or continuation therapy rather than *de novo* initiation in the ICU. Future studies should investigate whether early in-hospital statin initiation provides similar or additional benefits.

Demographic analysis highlights the importance of considering patient characteristics such as age and comorbidities when evaluating treatment efficacy, as patients in the statin group were generally older and had a higher prevalence of comorbid conditions known to complicate stroke management (Tu et al., 2024). Understanding these nuances can guide clinicians in identifying which patients are most likely to benefit from statin therapy, thereby enhancing personalized care strategies (Rieke et al., 1991).

Demographic analysis highlights the importance of considering patient characteristics such as age and comorbidities when evaluating treatment efficacy, as patients in the statin group were generally older and had a higher prevalence of comorbid conditions known to complicate stroke management (Tu et al., 2024). Understanding these nuances can guide clinicians in identifying which patients are most likely to benefit from statin therapy, thereby enhancing personalized care strategies (Rieke et al., 1991).

Subgroup analyses identified dementia as a potential effect modifier. Among patients with dementia, statin use was associated with a 45.2% mortality risk reduction (HR = 0.548, *P* = 0.034), with an NNT of 7 compared to 13 for non-dementia patients. The mechanistic basis for this enhanced benefit may involve statin-mediated inhibition of neuroinflammatory responses and reduction of cholesterol ester levels in nerve cells (van der Kant et al., 2019), as well as attenuation of Aβ-induced neurotoxicity through pathways independent of cholesterol lowering (Li et al., 2018). Additionally, statins may modulate cerebral blood flow autoregulation, potentially benefiting patients with vascular dementia (Zerin et al., 2025), and enhance atherosclerotic plaque stability, reducing stroke recurrence risk. However, given the ICD-based ascertainment of dementia in the primary cohort, this finding remains exploratory. The consistency of the effect direction in the external validation cohort, despite the interaction not reaching statistical significance, supports the plausibility of this association and warrants further investigation in larger cohorts with refined phenotypic definitions.

This study has several limitations. The retrospective design and reliance on data from a single database may restrict the generalizability of our findings. The lack of experimental validation raises questions about causality, and residual confounding from unmeasured variables cannot be excluded. Additionally, the current study did not evaluate the effect of statin dose on patient outcomes, and patient adherence information was lacking in the database. The sample size of the dementia subgroup was relatively small.

A further limitation concerns the ascertainment of dementia in the primary MIMIC-IV cohort, where ICD-based codes may not fully distinguish pre-existing dementia from acute encephalopathy, delirium, or post-stroke cognitive impairment in critically ill patients with impaired consciousness or aphasia. To mitigate this concern, we performed external validation using a cohort with clinically validated dementia diagnoses (as detailed in the section “Materials and methods). While the interaction did not reach statistical significance in the external cohort, the direction of effect remained consistent, suggesting that the observed effect modification warrants further investigation. Future prospective studies with standardized pre-stroke cognitive assessments and larger sample sizes are needed to confirm this exploratory finding.

In conclusion, baseline statin use is associated with significantly reduced short-to-medium-term mortality in critically ill ischemic stroke patients. The potential additional benefit observed in patients with dementia suggests that dementia status may be an effect modifier worthy of further investigation. However, given the observed time-dependent attenuation of the protective effect, timely statin use and ongoing evaluation of treatment strategies remain important. Prospective studies are needed to confirm these findings and to determine the optimal timing of statin initiation in the acute stroke setting.

## Conclusion

Statin therapy significantly reduces short- and long-term mortality (from 30 days to 1 year) among critically ill patients with ischemic stroke, and this protective effect was successfully replicated in the 30-days external validation. The interaction between statins and dementia did not reach statistical significance in the external validation. Given the inherent limitations of ICD-based dementia ascertainment in the primary cohort, the potential enhanced benefit observed in the dementia subgroup should be interpreted as an exploratory finding. Further prospective studies with standardized pre-stroke cognitive assessments are required to confirm whether dementia status truly modifies the survival benefit of statin therapy in critically ill stroke patients.

## Data Availability

The original contributions presented in this study are included in this article/Supplementary material, further inquiries can be directed to the corresponding author.
