# Supplementary material for: Statin use and all-cause mortality in critically ill ischemic stroke patients: a time-dependent Cox analysis with external validation highlighting enhanced survival benefits in dementia subgroups
Source: Front Aging Neurosci. 2026 Sep 15;18:1866416. doi: 10.3389/fnagi.2026.1866416 (PMC13619808; doi:10.3389/fnagi.2026.1866416)
Supplement: Supplementary file 1 [file Supplementary_file_1.docx]

Supplementary

Table S1. Baseline characteristics of the study cohorts

| **Characteristic** | **MIMIC-IV (n=1,964)** | **Clinical (n=4,255)** | **SMD** |
| --- | --- | --- | --- |
| **Demographics** |  |  |  |
| Age (years) | 68.3 ± 14.8 | 68.6 ± 14.5 | 0.025 |
| BMI (kg/m²) | 28.2 ± 6.7 | 28.7 ± 7.2 | 0.066 |
| Male, n (%) | 1,073 (54.6) | 2,152 (50.6) | 0.081 |
| **Comorbidities** |  |  |  |
| Hypertension | 1,259 (64.1) | 1,316 (30.9) | 0.704 |
| Diabetes | 658 (33.5) | 80 (1.9) | 0.911 |
| Heart failure | 568 (28.9) | 234 (5.5) | 0.653 |
| Chronic kidney disease | 406 (20.7) | 341 (8.0) | 0.367 |
| Dementia | 92 (4.7) | 82 (1.9) | 0.155 |
| **Exposure and Outcome** |  |  |  |
| Statin use, n (%) | 1,353 (68.9) | 1,451 (34.1) | 0.743 |
| 30-day mortality, n (%) | 431 (21.9) | 292 (6.9) | 0.44 |

Abbreviations: BMI, body mass index; SMD, standardized mean difference.

Note: SMD values > 0.1 indicate clinically meaningful differences between the cohorts. The observed differences in baseline comorbidities and statin utilization rates (SMD > 0.1) reflect inherent population heterogeneity between the MIMIC-IV and Clinical databases. All subsequent analyses were adjusted for these baseline characteristics to minimize confounding bias.

Table S2：Multivariable Cox Regression for Mortality in Severe Stroke

| **Variable** | **Timepoint** | **HR** | **CI** | **P Value** |
| --- | --- | --- | --- | --- |
| Statin therapy | 30-day | 0.405 | 0.334-0.491 | <0.001 |
| Age (per year) | 30-day | 1.029 | 1.021-1.036 | <0.001 |
| Male gender | 30-day | 1.072 | 0.883-1.302 | 0.48 |
| Black race | 30-day | 1.115 | 0.806-1.541 | 0.512 |
| Other race | 30-day | 1.855 | 1.516-2.269 | <0.001 |
| Statin therapy | 90-day | 0.48 | 0.404-0.571 | <0.001 |
| Age (per year) | 90-day | 1.024 | 1.018-1.031 | <0.001 |
| Male gender | 90-day | 0.994 | 0.838-1.180 | 0.947 |
| Black race | 90-day | 1.024 | 0.772-1.357 | 0.872 |
| Other race | 90-day | 1.72 | 1.435-2.061 | <0.001 |
| Statin therapy | 180-day | 0.525 | 0.445-0.620 | <0.001 |
| Age (per year) | 180-day | 1.024 | 1.018-1.030 | <0.001 |
| Male gender | 180-day | 1.017 | 0.864-1.197 | 0.843 |
| Black race | 180-day | 1.011 | 0.776-1.318 | 0.934 |
| Other race | 180-day | 1.643 | 1.380-1.955 | <0.001 |
| Statin therapy | 1-year | 0.536 | 0.459-0.627 | <0.001 |
| Age (per year) | 1-year | 1.023 | 1.017-1.028 | <0.001 |
| Male gender | 1-year | 1.01 | 0.866-1.177 | 0.903 |
| Black race | 1-year | 0.986 | 0.770-1.262 | 0.912 |
| Other race | 1-year | 1.626 | 1.378-1.917 | <0.001 |

Table S3 Comparison of study outcomes between the primary MIMIC-IV cohort and the external validation Clinical cohort

| **Variable** | **MIMIC-IV Primary Cohort (n=1,964)** | **Clinical Validation Cohort (n=4,255)** |
| --- | --- | --- |
| Adjusted HR (95% CI) for 30-day mortality | 0.406 (0.314–0.955) | 0.622 (0.476–0.811) |
| Statin-Dementia interaction HR (95% CI) | 0.548 (0.314–0.955) | 0.579 (0.068–4.962) |
| P-value for Statin-Dementia interaction | 0.034 | 0.44 |
| Sample size (Total / Dementia) | 1,964 / 92 | 4,255 / 82 |

Abbreviations: HR, hazard ratio; CI, confidence interval.

The primary effect of statin therapy on 30-day mortality was successfully validated in the Clinical cohort. Although the subgroup effect of statin in dementia patients showed a consistent direction, the interaction test did not reach statistical significance, likely due to the limited sample size and number of events in the dementia subgroup within the Clinical database.

Table S4：Results of the main subgroup analysis

| **Time Point** | **Subgroup** | **HR** | **Risk Reduction** |
| --- | --- | --- | --- |
|  |  |  |  |
| 30-day | Overall | 0.405 | 59.50% |
| 30-day | Race_OTHER | 0.428 | 57.20% |
| 30-day | Age_75-84 | 0.323 | 67.70% |
| 30-day | Dementia | 0.229 | 77.10% |
| 30-day | Hypertension | 0.392 | 60.80% |
| 30-day | Diabetes | 0.43 | 57.00% |
| 90-day | Overall | 0.48 | 52.00% |
| 90-day | Race_OTHER | 0.477 | 52.30% |
| 90-day | Age_75-84 | 0.402 | 59.80% |
| 90-day | Dementia | 0.224 | 77.60% |
| 90-day | Hypertension | 0.453 | 54.70% |
| 90-day | Diabetes | 0.518 | 48.20% |
| 180-day | Overall | 0.525 | 47.50% |
| 180-day | Race_OTHER | 0.513 | 48.70% |
| 180-day | Age_75-84 | 0.462 | 53.80% |
| 180-day | Dementia | 0.27 | 73.00% |
| 180-day | Hypertension | 0.509 | 49.10% |
| 180-day | Diabetes | 0.593 | 40.70% |
| 1-year | Overall | 0.536 | 46.40% |
| 1-year | Race_OTHER | 0.524 | 47.60% |
| 1-year | Age_75-84 | 0.456 | 54.40% |
| 1-year | Dementia | 0.261 | 73.90% |
| 1-year | Hypertension | 0.539 | 46.10% |
| 1-year | Diabetes | 0.584 | 41.60% |

Table S5: Results of Interaction Between Statin Treatment and Subgroups in Patients with Severe Stroke

| **Time point** | **Comorbidity** | **Interaction Term** | **Coef** | **HR** | **P Value** |
| --- | --- | --- | --- | --- | --- |
| 30d | congestive_heart_failure | statin_num:congestive_heart_failure1 | 0.2159 | 1.241 | 0.307 |
| 30d | renal_disease | statin_num:renal_disease1 | 0.273 | 1.314 | 0.284 |
| 30d | diabetes | statin_num:diabetes1 | 0.0516 | 1.053 | 0.812 |
| 30d | hypertension | statin_num:hypertension1 | -0.1869 | 0.83 | 0.349 |
| 30d | cerebrovascular_disease | statin_num:cerebrovascular_disease1 | -0.069 | 0.933 | 0.868 |
| 30d | dementia | statin_num:dementia1 | -0.616 | 0.54 | 0.093 |
| 90d | congestive_heart_failure | statin_num:congestive_heart_failure1 | 0.1349 | 1.144 | 0.474 |
| 90d | renal_disease | statin_num:renal_disease1 | 0.3403 | 1.405 | 0.131 |
| 90d | diabetes | statin_num:diabetes1 | 0.0478 | 1.049 | 0.807 |
| 90d | hypertension | statin_num:hypertension1 | -0.2496 | 0.779 | 0.157 |
| 90d | cerebrovascular_disease | statin_num:cerebrovascular_disease1 | -0.2949 | 0.745 | 0.394 |
| 90d | dementia | statin_num:dementia1 | -0.774 | 0.461 | 0.015 |
| 180d | congestive_heart_failure | statin_num:congestive_heart_failure1 | 0.1272 | 1.136 | 0.482 |
| 180d | renal_disease | statin_num:renal_disease1 | 0.4084 | 1.504 | 0.059 |
| 180d | diabetes | statin_num:diabetes1 | 0.1076 | 1.114 | 0.565 |
| 180d | hypertension | statin_num:hypertension1 | -0.2122 | 0.809 | 0.209 |
| 180d | cerebrovascular_disease | statin_num:cerebrovascular_disease1 | -0.2724 | 0.762 | 0.383 |
| 180d | dementia | statin_num:dementia1 | -0.6377 | 0.529 | 0.033 |
| 1y | congestive_heart_failure | statin_num:congestive_heart_failure1 | 0.0767 | 1.08 | 0.653 |
| 1y | renal_disease | statin_num:renal_disease1 | 0.3103 | 1.364 | 0.118 |
| 1y | diabetes | statin_num:diabetes1 | 0.0504 | 1.052 | 0.772 |
| 1y | hypertension | statin_num:hypertension1 | -0.1203 | 0.887 | 0.449 |
| 1y | cerebrovascular_disease | statin_num:cerebrovascular_disease1 | -0.1203 | 0.887 | 0.64 |
| 1y | dementia | statin_num:dementia1 | -0.6551 | 0.519 | 0.02 |
| **Timepoint** | **Subgroup** | **Interaction_Term** | **Coef** | **HR** | **P Value** |
| 30d | age_group | statin_num:age_group65-74 | 0.1527 | 1.165 | 0.575 |
| 30d | age_group | statin_num:age_group75-84 | -0.187 | 0.829 | 0.497 |
| 30d | age_group | statin_num:age_group≥85 | 0.0756 | 1.079 | 0.795 |
| 30d | gender | statin_num:genderM | 0.1054 | 1.111 | 0.587 |
| 30d | race | statin_num:raceBLACK | 0.0444 | 1.045 | 0.896 |
| 30d | race | statin_num:raceOTHER | -0.1168 | 0.89 | 0.571 |
| 90d | age_group | statin_num:age_group65-74 | 0.1138 | 1.12 | 0.64 |
| 90d | age_group | statin_num:age_group75-84 | -0.1389 | 0.87 | 0.56 |
| 90d | age_group | statin_num:age_group≥85 | -0.0339 | 0.967 | 0.897 |
| 90d | gender | statin_num:genderM | -0.0016 | 0.998 | 0.993 |
| 90d | race | statin_num:raceBLACK | -0.0797 | 0.923 | 0.793 |
| 90d | race | statin_num:raceOTHER | -0.2245 | 0.799 | 0.227 |
| 180d | age_group | statin_num:age_group65-74 | 0.2247 | 1.252 | 0.339 |
| 180d | age_group | statin_num:age_group75-84 | 0.0086 | 1.009 | 0.97 |
| 180d | age_group | statin_num:age_group≥85 | 0.071 | 1.074 | 0.778 |
| 180d | gender | statin_num:genderM | 0.0302 | 1.031 | 0.856 |
| 180d | race | statin_num:raceBLACK | -0.0811 | 0.922 | 0.78 |
| 180d | race | statin_num:raceOTHER | -0.2456 | 0.782 | 0.171 |
| 1y | age_group | statin_num:age_group65-74 | 0.2821 | 1.326 | 0.203 |
| 1y | age_group | statin_num:age_group75-84 | 0.0192 | 1.019 | 0.929 |
| 1y | age_group | statin_num:age_group≥85 | 0.1483 | 1.16 | 0.535 |
| 1y | gender | statin_num:genderM | 0.0213 | 1.021 | 0.892 |
| 1y | race | statin_num:raceBLACK | 0.0429 | 1.044 | 0.877 |
| 1y | race | statin_num:raceOTHER | -0.2203 | 0.802 | 0.193 |

Table S6：Baseline Characteristics by Dementia Status

| **Characteristic** | **Overall** | **0** | **1** | **p-value^2^** |
| --- | --- | --- | --- | --- |
|  | **N = 1,964^1^** | **N = 1,872^1^** | **N = 92^1^** |  |
| age | 68.27 (14.78) | 67.65 (14.73) | 80.93 (8.86) | <0.001 |
| gender |  |  |  | 0.2 |
| F | 891 (45%) | 843 (45%) | 48 (52%) |  |
| M | 1,073 (55%) | 1,029 (55%) | 44 (48%) |  |
| race |  |  |  | >0.9 |
| ASIAN | 58 (3.0%) | 56 (3.0%) | 2 (2.2%) |  |
| BLACK | 229 (12%) | 218 (12%) | 11 (12%) |  |
| OTHER | 516 (26%) | 493 (26%) | 23 (25%) |  |
| WHITE | 1,161 (59%) | 1,105 (59%) | 56 (61%) |  |
| sofa | 4.95 (3.47) | 4.93 (3.48) | 5.39 (3.32) | 0.093 |
| sapsii | 38.00 (13.79) | 37.69 (13.70) | 44.27 (14.14) | <0.001 |
| oasis | 34.06 (8.82) | 33.85 (8.77) | 38.41 (8.72) | <0.001 |
| sirs |  |  |  | 0.1 |
| 0 | 33 (1.7%) | 31 (1.7%) | 2 (2.2%) |  |
| 1 | 253 (13%) | 245 (13%) | 8 (8.7%) |  |
| 2 | 596 (30%) | 557 (30%) | 39 (42%) |  |
| 3 | 723 (37%) | 692 (37%) | 31 (34%) |  |
| 4 | 359 (18%) | 347 (19%) | 12 (13%) |  |
| gcs | 12.88 (3.35) | 12.92 (3.33) | 12.11 (3.59) | 0.023 |
| renal_disease |  |  |  | 0.11 |
| 0 | 1,558 (79%) | 1,491 (80%) | 67 (73%) |  |
| 1 | 406 (21%) | 381 (20%) | 25 (27%) |  |
| diabetes |  |  |  | 0.021 |
| 0 | 1,306 (66%) | 1,255 (67%) | 51 (55%) |  |
| 1 | 658 (34%) | 617 (33%) | 41 (45%) |  |
| hypertension |  |  |  | 0.12 |
| 0 | 705 (36%) | 679 (36%) | 26 (28%) |  |
| 1 | 1,259 (64%) | 1,193 (64%) | 66 (72%) |  |
| statin |  |  |  | >0.9 |
| 0 | 611 (31%) | 582 (31%) | 29 (32%) |  |
| 1 | 1,353 (69%) | 1,290 (69%) | 63 (68%) |  |
| ^1^ Mean (SD); n (%) | | | | |
| ^2^ Wilcoxon rank sum test; Pearson’s Chi-squared test; Fisher’s exact test | | | | |

Figure S1 Baseline Characteristics Balance Before and After IPTW Weighting

Note:The heatmap displays the standardized mean differences (SMDs) of key baseline characteristics before and after IPTW weighting. The color intensity indicates the degree of balance, ranginfrom green (excellent balance, SMD < 0.1) to red (severe imbalance, SMD ≥ 0.5). The numerical values represent the secific SMDs. Post-weighting, all variables exhibit SMDs < 0.1, indicating successful achievement of good balance between groups.

Figure S2 Forest plot of multi-method validation

Note:The figure shows that the effect directions of all methods are highly consistent.

Figure S3 Time-dependent protective effect of statin treatment

Note:The figure shows that the protective effect weakens over time.

Figure S4 Clinical impact of statin therapy: Number needed to treat (NNT)

Note:Compared to patients without dementia, patients with dementia have a smaller NNT.

Figure S5 Forest plot of subgroup analyses for the association between statin use and 30-day mortality in the external validation cohort (clinical)

Hazard ratios (HRs) and 95% confidence intervals (CIs) were estimated using Cox proportional hazards models. For subgroups other than age and sex, models were adjusted for age and sex. The vertical dashed line indicates HR = 1 (no effect). Overall HR for statin (multivariable‑adjusted) was 0.622 (95% CI: 0.476‑0.811). The interaction between statin and dementia was not statistically significant (p for interaction = 0.44). The wide confidence interval in the dementia subgroup reflects the limited number of patients with dementia (n = 82) and few events, which precludes a definitive conclusion regarding effect modification.
